# The potential therapy with dental tissue-derived mesenchymal stem cells in Parkinson’s disease

**DOI:** 10.1186/s13287-020-01957-4

**Published:** 2021-01-06

**Authors:** Zhuangzhuang Xiao, Tong Lei, Yanyan Liu, Yanjie Yang, Wangyu Bi, Hongwu Du

**Affiliations:** 1 112 Lab, School of Chemistry and Biological Engineering, University of Science and Technology Beijing, 30 XueYuan Road, Haidian District, Beijing, 100083 China; 2 Kangyanbao (Beijing) Stem Cell Technology Co., Ltd, Beijing, 102600 China

**Keywords:** Cell therapy, Dopaminergic neurons, Dental pulp stem cells, Parkinson’s disease, Stem cells from human exfoliated deciduous teeth

## Abstract

Parkinson’s disease (PD), the second most common neurodegenerative disease worldwide, is caused by the loss of dopaminergic (DAergic) neurons in the substantia nigra resulting in a series of motor or non-motor disorders. Current treatment methods are unable to stop the progression of PD and may bring certain side effects. Cell replacement therapy has brought new hope for the treatment of PD. Recently, human dental tissue-derived mesenchymal stem cells have received extensive attention. Currently, dental pulp stem cells (DPSCs) and stem cells from human exfoliated deciduous teeth (SHED) are considered to have strong potential for the treatment of these neurodegenerative diseases. These cells are considered to be ideal cell sources for the treatment of PD on account of their unique characteristics, such as neural crest origin, immune rejection, and lack of ethical issues. In this review, we briefly describe the research investigating cell therapy for PD and discuss the application and progress of DPSCs and SHED in the treatment of PD. This review offers significant and comprehensive guidance for further clinical research on PD.

## Introduction

Parkinson’s disease (PD) is the second most common neurodegenerative disease in the world after Alzheimer’s disease with incidences of 1% and 5% for populations aged over 65 and 80, respectively [1]. One of the main pathological features of PD is that the loss of DAergic neurons in the *substantia nigra pars compacta* (SNpc) leads to a significant decrease in the content of dopamine (DA) in the striatum, and there are Lewy bodies with α-synuclein as the main component in the surviving neurons [2, 3]. The loss of these neurons will lead to some clinical symptoms related to the disease, such as static tremor, bradykinesia, rigidity, and postural gait disorders, along with other non-motor symptoms [4]. Although the exact pathogenesis of PD is still uncertain, it has been reported that mitochondrial dysfunction, oxidative stress, neuroinflammation, and environmental toxins are important factors for the death of DAergic neurons [5, 6].

At present, drug therapy is the most effective and widely used treatment for PD patients, including administration of levodopa, DA agonists, amantadine, monoamine oxidase B (MAO-B) inhibitors [7], catechol-O-methyltransferase (COMT) inhibitors [8], and some anticholinergic drugs. As physiotherapy, nucleus destruction and deep brain stimulation (DBS) [9] are new and effective methods, which have great potential for popularization and application. In addition, some adjuvant therapies also are effective for remission and partial treatment of patients with PD. Although these treatments have improved certain symptoms of the disease to some extent, they have not prevented the progression of PD and also cause some side effects. In recent years, cell transplantation has been considered to be a new option for the treatment of neurodegenerative diseases [10, 11]. Stem cells are widely used in PD to counteract the harmful effects of DAergic neuron loss, because of their high proliferative capacity and multi-lineage differentiation potential.

Of all available stem cell sources, human dental tissue-derived mesenchymal stem cells (such as DPSCs and SHED) not only feature the universal characteristics of stem cells but have also attracted increasing attention from PD researchers for their neural crest origin, immunomodulatory activity, and non-tumorigenic properties [10] and for avoidance of ethical problems caused by transplantation [12, 13]. Owing to the neurocrest origin of DPSCs and SHED, they achieve previously unimagined capability for treating central nervous system diseases and peripheral nerve injuries such as caries and alveolar bone atrophy [14, 15]. DPSCs and SHED can play a powerful role in the treatment of PD. They can be differentiated into DAergic neuron-like cells and secrete neurotrophic factors such as brain-derived neurotrophic factor (BDNF) and glial cell line-derived neurotrophic factor (GDNF) [16–18]. DPSCs and SHED have high proliferation ability, immunomodulatory characteristics, neurodifferentiation ability, and non-ethical and material advantages, which make them potential clinical therapeutic materials for PD. These cells are gradually becoming the priority of researchers in the cell therapy of PD. In recent years, DPSCs and SHED have been applied to the preclinical study of PD (Table 1). So far, there is no comprehensive overview of the application of DPSCs and SHED in the treatment of PD. Therefore, this review briefly describes the research course of cell therapy for PD and reports the application and research progress of DPSCs and SHED in the treatment of PD.

**Table 1:** Experimental study on the therapeutic role of DPSCs and SHED in PD models

Type	Study model	Delivery method	Sample size (***n***)	Number of cells	Treatment duration	Survival time	Neuron improvement	Behaviour test	Mechanism	Authors, year
DPSCs	MPTP-induced Swiss albino mice	IN	20	5 × 10^5^	4 weeks	> 4 weeks	TH	Beam traversal test; cylinder test; adhesive removal test	Repair neurogenic niche	Simon et al., 2019 [19]
DPSCs	Co-culture system of neurons and microglia induced with MPTP	Co-culture	–	5 × 10^3^	2 days	**–**	Microglia	**–**	Against neuroinflammation	Gnanasegaran et al., 2017 [20]
DPSCs	MPTP-induced Old Swiss albino mice	IT	45	1 × 10^6^	12 weeks	–	TH	Beam traversal test; cylinder test; adhesive removal test; Buried pellet test; Block test	Immuno-modulation and neuro-restoration	Gnanasegaran et al., 2017 [10]
DPSCs	Mesencephalic cells of CD1 mice induced with MPP^+^ or rotenone	Co-culture	–	5 × 10^3^	2 days	–	DA	**–**	Paracrine, secretion of neurotrophic factors	Nesti et al., 2011 [16]
Exosome of SHED	6-OHDA-induced Wistar rats	IN	16	43 × 10^8^	17 days	–	TH	Apomorphine-induced rotation behaviour test; CatWalk gait test	Anti-oxidative stress	Narbute et al., 2019 [21]
SHED	6-OHDA-induced Wistar rats	IC	17	5 × 10^5^	16 weeks	About 16 weeks	DA	Apomorphine-induced rotation behaviour test; rotarod test	Immuno-modulation and neuro-restoration	Zhang et al., 2018 [22]
Exosome of SHED	ReNcell VM human neural stem cells induced with 6-OHDA	Co-incubation	–	–	20 h	–	DA	–	Anti-oxidative stress	Jarmalavičiūtė et al., 2015 [17]
SHED	6-OHDA-induced Sprague-Dawley rats	IC	14	2 × 10^5^	6 weeks	> 6 weeks	DA; TH; CGNs	Methamphetamine-induced rotation test	Paracrine, secretion of neurotrophic factors	Fujii et al., 2015 [23]
SHED	6-OHDA-induced Sprague-Dawley rats	IC	24	5 × 10^5^	8 weeks	> 6 weeks	DA	Apomorphine-induced rotation behaviour test	Neuro-restoration and paracrine effect	Wang et al., 2010 [24]

*DPSCs* dental pulp stem cells, *SHED* stem cells from human exfoliated deciduous teeth, *MPTP* 1-methyl-4-phenyl-1,2,3,6-tetrahydropyridine, *MPP*^+^ 1-methyl-4-phenylpyridinium, *6-OHDA* 6-hydroxydopamine, *IN* intranasal, *IT* intrathecal, *IC* intracerebral, *TH* tyrosine hydroxylase, *DA* dopamine, *CGNs* cerebellar granule neurons

## Cell therapy for PD

PD research has always pioneered cell transplantation therapy because of the unique pathological characteristics—loss of DAergic neurons. Since the 1980s, researchers have been trying to save the lost DAergic neurons by cell transplantation [25]. Initially, a variety of catecholaminergic cells were selected [26], but the most successful method was to use tissue dissected from the developing foetal midbrain. However, although this method has proved successful in experiments, the clinical effect is not satisfactory. This is mainly due to the following reasons: (1) ethical problems are inherent in the use of human foetal tissue, (2) there are practical problems caused by the need for sufficient foetal tissue for each patient, and (3) inconsistent results and side effects of dyskinesia were obtained in the “TRANSEURO” study [9, 27, 28]. Therefore, in order to find more easily available sources of substantia nigra DA cells for transplantation, researchers have studied dopaminergic neurons in different species and have begun to explore different types of stem cells. At present, this field has been developed to produce a large number of substantia nigra DA cells safely and effectively from different types of stem cells. Compared with foetal tissue cell transplantation, stem cells play an irreplaceable role for the following reasons: (1) better availability [29], (2) standardized production [30], (3) controllable cell characteristics before transplantation (unfavourable consequences can be avoided [31, 32]), (4) good preservation conditions [30], and (5) precise preoperative control [9], including of dose, concentration, and volume. Therefore, these properties further promote the favourable prospect for stem cell transplantation in PD and inspire researchers to begin to treat the disease through cellular reprogramming.

With the development of stem cell therapy, many types of cells have been used for derivation and differentiation of DAergic neurons, drug screening, and cell therapy for PD [33]. At present, embryonic stem cells (ESCs), neural stem cells (NSCs), induced pluripotent stem cells (iPSCs), and mesenchymal stem cells (MSCs) are considered to be reliable cell sources for PD therapy [28, 34, 35]. To better understand the efficacy and safety of cell replacement therapy, a variety of experimental models of PD have been established. Toxicity-inducing drugs include 1-methyl-4-phenyl-1,2,3,6-tetrahydropyridine (MPTP), 6-hydroxydopamine (6-OHDA), and rotenone. These drugs act on cells or animal models to simulate the pathological characteristics of PD. Next, transfer of differentiated or undifferentiated ESCs, NSCs, iPSCs, and other types of stem cells into an animal model can restore a number of lost neurons or improve the behaviour of the animal model to varying degrees.

ESCs were once thought to constitute the best source of cells such as substantia nigra DAergic neurons [36]. It has been known for a long time that undifferentiated ESCs can be smoothly transplanted into the striatum of a rodent and have the ability to differentiate into DAergic neurons, which slows progression of behavioural abnormality in PD rat models [37, 38]. It is a good choice to induce more DAergic neurons in vitro, so the percentage of DAergic neurons can be greatly increased by inhibiting certain molecular pathways [39, 40] and/or using developmental factors related to DA formation, such as Sonic hedgehog, fibroblast growth factor 8, and brain-derived neurotrophic factor. These derived neurons can also be transplanted to experimental animal models and achieve unexpected benefits. However, the same outstanding problem as with iPSCs is that the tumorigenicity after transplantation cannot effectively be solved. In addition, ESC transplantation also involves certain ethical issues. There are more-or-less unsolved problems regarding the safety and effectiveness of other stem cell transplantation approaches which are not discussed in detail in this review. It is worth noting that a breakthrough clinical trial was launched in Japan in 2018 to transplant allogeneic human iPSC-derived dopaminergic neuronal precursors into the striatum of patients with PD [9, 41]. Further clinical trials of cell transplantation in the treatment of PD are expected to begin soon.

**Fig. 1:**
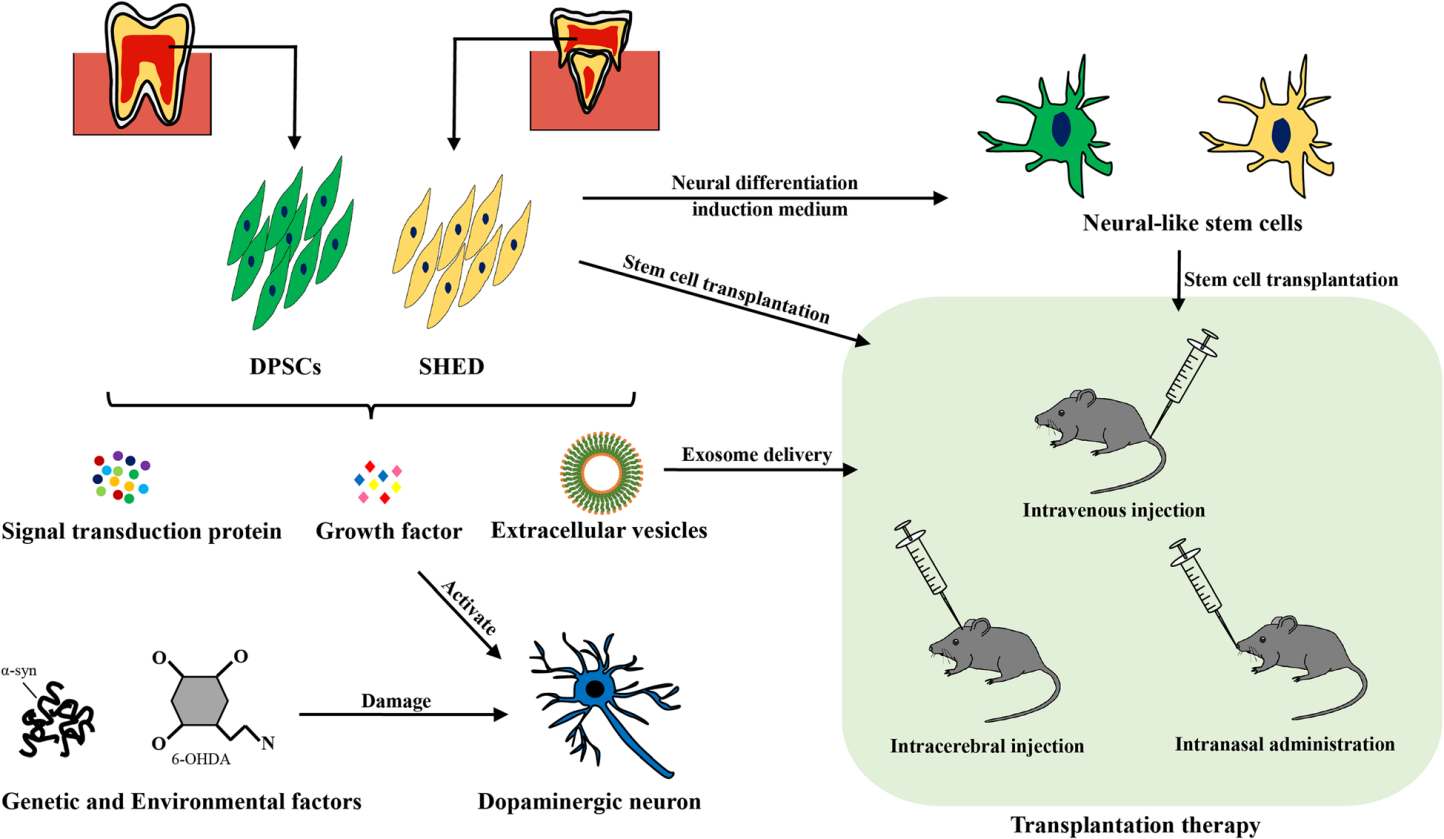
Main ways to exert the curative effects of DPSCs and SHED in PD models. Both genetic factors and environmental toxins can cause damage to DAergic neurons and then induce PD. DPSCs and SHED can be isolated from the tissue of teeth. On the one hand, DPSCs and SHED can be directly transplanted into PD animal models for treatment through different delivery methods. On the other hand, DPSCs and SHED can be induced to differentiate into DAergic neuron-like cells in vitro and then be transplanted in several ways. In addition, DPSCs and SHED can secrete signal transduction proteins, nerve growth factors, and extracellular vesicles to repair damaged neurons. Extracellular vesicles are mainly composed of microvesicles and exosomes. Exosomes of appropriate size can also be transferred to the animal model by several delivery methods. There are many means to transplant, such as intravenous injection, intranasal injection, and intracerebral injection

Mesenchymal stem cells (MSCs) are a type of adult stem cells isolated from a variety of tissues [42]. MSCs have the potential for multi-directional differentiation, such as with osteogenesis, adipogenesis, and neuroblast formation, and have a unique function of secreting cytokines, which has been used in a number of clinical trials. At present, eight unique MSCs [43] have been isolated from different dental tissues and used in the treatment of various diseases. DPSCs and SHED have proved to be promising potential options for the treatment of PD by multiple modes of action (Fig. 1). Next, we will discuss the current application and progress of DPSCs and SHED in the treatment of PD.

## Dental pulp stem cells therapy for PD

### Characteristics of DPSCs

DPSCs were first discovered by *Gronthos* and colleagues through the study of dental pulp cells in 2000 [44]. DPSCs share similar characteristics with bone marrow mesenchymal stem cells (BMSCs), such as the morphology of fibroblasts and the ability to form colonies in vitro. They can express many surface markers such as CD73, CD90, and CD105 but do not express surface markers such as CD14, CD34, and CD 45 [44–46]. However, compared with BMSCs, DPSCs exhibit a higher proliferation rate, more exuberant colony and colonies formation ability, and stronger mineralization potential [44, 47, 48]. In addition, DPSCs can also express the ESCs markers OCT4, SOX2, and MYC, which are not common in MSCs [49]. Similarly, in addition to odontogenic potential, DPSCs also have potential for multi-lineage differentiation. In vitro, DPSCs can be differentiated into various types of cells, such as adipocytes, hepatocytes, osteoblasts, and neuronal cells under suitable conditions. Additionally, there is sufficient evidence to show their immunomodulatory properties and the capacity of ectopic formation in vivo [43].

DPSCs are stem cells derived from ectoderm, which originate from migrated neural crest cells and exhibit strong plasticity. They can express markers of nerve lineage [50, 51], such as low-affinity nerve growth factor receptor p75, intermediate filament nestin, and glial fibrillary acidic protein (GFAP), and more mature markers of nerve lineage such as β-III tubulin and nuclear antigen, which also reflects their origin and high heterogeneity [52, 53]. Of course, a large number of studies have shown that it can differentiate into DAergic neuron-like cells [10, 16, 54, 55]. In addition, DPSCs can also produce and secrete neurotrophic factors, induce axon guidance, and differentiate into functionally active neurons, which also illustrates great potential for cell transplantation therapy for PD.

### Preclinical application of DPSCs in Parkinson’s disease

The neural differentiation of DPSCs has been studied in vitro. According to the cell morphology and the expression of early neuronal markers, it has been suggested that these cells can differentiate into neurons [46, 52, 54, 56, 57]. In vivo studies have further shown that DPSCs can survive and express neuronal markers after transplantation into the brain [58]. This potential for spontaneous differentiation of DPSCs also convincingly proves its role in treatment by nerve regeneration. With cell therapy for PD, from the initial transplantation of foetal midbrain tissue to the study of animal models and clinical trials with PD patients, research has provided us with proof-of-concept evidence that transplantation of DAergic neuroblasts into the striatum can effectively relieve the symptoms of PD. We mentioned above that DPSCs can differentiate into dopaminergic neuron-like cells and secrete neurotrophic factors (Fig. 1). For example, *Claudia* [10] studied the neuroprotective effect of DPSCs using DPSCs and a midbrain cell indirect co-culture system and proved for the first time the protective effect of DPSCs on dopaminergic neurons in PD models induced by MPP^+^ or rotenone. It is speculated that their neuroprotective effects may be caused by soluble factors released by DPSCs, such as brain-derived neurotrophic factor (BDNF) and nerve growth factor (NGF). Therefore, in PD cell therapy, the differentiation of stem cells into DAergic neurons is not the only purpose, but the ability to produce and secrete neuroprotective factors may be more important [11]. These secreted neuroprotective factors may bind to specific receptors and trigger the activation of certain signalling pathways that coordinate cell function.

Transplanting DPSCs directly into the brain of an animal model of PD does not seem to be a very effective treatment. At present, a nerve-inducing mixture of various chemicals and cytokines is widely used to cause DPSCs to progress towards the fate of neurogenesis in vitro (Fig. 1). The neuronal differentiation of DPSCs basically consists of two steps: the addition of medium for epigenetic reprogramming and neural induction and subsequent neuronal maturation. One study differentiated DPSCs into DAergic neuron-like cells after five stages [54]. After the formation of neurospheres in the second stage, they were cultured in N-2 medium containing a mixture of nerve inducers including fibroblast growth factor (FGF), FGF-8b, sonic hedgehog-N, and ascorbic acid, and the differentiation rate of DAergic neuron-like cells was 14.49% (refers to the ratio of tyrosine hydroxylase positive cells). This method not only has a low differentiation rate but is also time-consuming. Some reports have shown that DPSCs can differentiate into functional DAergic neurons [59–61]. However, other researchers have found that they may only differentiate into premature DAergic neurons rather than functional and mature DAergic neurons [62]. These contradictory findings led a team recently to study the differentiation into dopaminergic neurons of DPSCs under different chemical and photobiological regulation conditions [55]. Their results showed that photobiological regulation can increase the expression of dopaminergic neuron protective protein mRNAs in DPSCs under certain conditions. However, DPSC-induced DAergic neurons have the characteristics of immature neurons and are prone to death. The reason for the analysis may be that there is no cell niche including other cell types and extracellular matrix structure in vitro. For now, the research on the treatment of PD by inducing DPSCs to differentiate into DAergic neurons still needs improvement.

Any injury can cause inflammation. Neuroinflammation is one of the many pathogenic mechanisms that lead to the death of DAergic neurons in the substantia nigra of patients with PD. Autopsy studies have basically confirmed the existence of innate immunity and adaptive immunity in the injured brain regions of patients with PD [63]. Activated microglia and T lymphocytes can be detected in the substantia nigra of patients, and increased expression of pro-inflammatory mediators can also be detected [64, 65]. Thus, a perfect cell transplantation programme should include suppressing inflammation in addition to maintaining the number of neurons, which DPSCs can do. In vitro, inflammation can be achieved by a variety of methods, including the use of MPTP or LPS for induction. Both toxins can inhibit the proliferation of DAergic neurons and enhance the expression of inflammatory mediators. At the same time, microglia clearly proliferate, which leads to the co-activation of TLR4 and IFN- γ receptors and initiates the mechanism of nerve cell death [66]. A study was conducted to investigate the effects of DPSCs in an inflammatory microenvironment when neurons and microglia were exposed to the neurotoxin MPTP to observe the neuroimmunomodulatory properties of DPSCs in an in vitro PD model [20]. The results showed that DPSCs could significantly inhibit the production of ROS and NO and regulate the levels of pro-inflammatory factors such as IL-1α, IL-1β, IFN-γ, and TNF-α. In addition, although DPSCs were exposed to environmental toxins, they were still able to express neuronal markers such as Nestin, Pax6, and Nurr1. At the same time, similar results were obtained in another in vivo experiment in which the behavioural defects of PD mice were reversed to some extent [10]. In both studies, elevated levels of anti-inflammatory factors IL-13, IL-4, IL-10, and TNF-α were observed, which can neutralize the inflammatory process. Some researchers speculate that these factors can be secreted by neurons or microglia, and they may reduce inflammation by promoting the M2 microglia phenotype and causing death of the M1 microglia phenotype or by enhancing the effects of oxidative stress on neurons through the JAK/STAT pathway [67]. We tend to hold that these secreted factors originate from the transplanted DPSCs. This paracrine process of producing and secreting various nutritional factors may be the main mechanism by which DPSCs play a role in the treatment of PD.

However, one of the challenges is to find a safe and effective method of cell delivery before DPSC transplantation is used in clinical applications. It has been reported that the survival rate of transplanted cells, the full enrichment of therapeutic cells in the brain, and success in avoiding the distribution of stem cells to peripheral organs are all affected by transplantation methods [28, 68]. Due to the existence of the blood-brain barrier (BBB), DPSC transplantation is also faced with the problem of invasiveness and invalidity. However, some studies have explored the nasal system as a new pathway for stem cell delivery, which can bypass the BBB and directly target brain therapy for PD [69, 70]. Therefore, a recent study showed that degenerated tyrosine hydroxylase (TH)-positive neurons, motor coordination, and olfactory function were significantly improved by intranasal administration of PKH26 pre-labelled DPSCs into MPTP-induced PD mice [19]. In another study, intrathecal injection also significantly promoted the recovery of neurons and the improvement of behavioural function [10]. Comparatively, the method of intrathecal injection may be cumbersome and difficult. Regardless of how the DPSCs are administered, the ability of DPSCs to differentiate into DAergic neurons in vivo or in vitro and its positive role in PD therapy have been convincingly confirmed.

## Treatment of PD with stem cells from human exfoliated deciduous teeth

### Characteristics of SHED

Stem cells from human exfoliated deciduous teeth (SHED) constitutes a unique population of pluripotent stem cells first isolated from the residual pulp of deciduous teeth by Miura et al. in 2003 [46]. They also have the characteristics of MSCs, but compared with DPSCs and BMSCs, SHED exhibit a stronger proliferation rate, higher cell population doubling rate, and more vigorous ability to form spherical cell clusters. The surface labelling of SHED meets the minimum standard proposed by ISCT. They express CD13, CD29, CD44, CD56, CD73, CD90, CD105, CD146, and CD166 but not CD14, CD19, CD24, CD31, CD34, CD45, CD117, or CD133 [46, 71–74]. SHED are also derived from migrated neural crest cells. They can express the neural stem cell marker nestin, embryonic stem cell markers Oct4 and Nanog, and stage-specific embryonic antigens SSEA-3 and SSEA-4 [46]. Additionally, SHED has higher levels of basic fibroblast growth factor (bFGF) and bone morphogenic protein 2 (BMP-2) gene expression than do BMSCs and DPSCs [75]. In addition, the favourable immunomodulatory properties of SHED have been fully proven in the research of related diseases [76].

SHED has highly potential for multi-directional differentiation. In addition to odontogenic potential, they can differentiate into osteogenic, adipogenic, hepatogenic, and neurogenic lineage cells under suitable conditions in vitro. Under the condition of neural induction culture, SHED has been found to express the early neural marker nestin, the late neural marker neuron-specific enolase (NSE), glial fibrillary acidic protein (GFAP), and 2′,3′-cyclic nucleotide 3′-phosphodiesterase (CNP) [77]. It has been reported that transplantation of undifferentiated or nerve-induced SHED into a rat spinal cord injury (SCI) model can promote functional recovery [78]. This indicates the potential of implanted SHED or its derivatives in the treatment of SCI in rodents and other neurodegenerative diseases. In addition, a large number of studies on the culture of SHED-derived neurospheres and the induction of dopaminergic neurons as well as the treatment of PD rat models have shown that they constitute a very suitable cell source for cell therapy in PD [22–24].

### Preclinical application of SHED in Parkinson’s disease

Previous studies on the neural potential of DPSCs and SHED without neural induction have shown that these cells can express nestin, a neural progenitor cell marker, and GFAP, a glial cell marker, at the mRNA and protein levels [45, 46]. In vitro differentiation studies have also shown that they can differentiate into nerve cells and can survive and express neuronal markers when SHED is transplanted into the brain of adult rodents. In addition, they are neural crest-derived cells and are easy to obtain, which further indicates their potential application value for the treatment of PD.

A recent study explored the efficiency of SHED for differentiation into DAergic neuron-like cells and the ability of DAergic neuron-like cells to secrete dopamine and evaluated the therapeutic effect when SHED were transplanted into a 6-OHDA-induced (nigrostriatal damage mainly caused by oxidative stress) PD rat model [23]. The team used a hypoxia-induced differentiation scheme to effectively differentiate SHED into DAergic neuron-like cells. After undifferentiated and differentiated SHED were transplanted into the PD rat model, behavioural disorder and the number of TH-positive cells were significantly improved, and the protective effect on endogenous DAergic neurons was also observed. This subject also demonstrates that the paracrine mechanism during this differentiation helps to combat 6-OHDA-induced neurodegeneration and repair nigrostriatal damage, confirming the conclusions of previous studies showing that these dental pulp-derived stem cells promote the functional recovery of various acute and chronic central nervous system injuries through cell replacement and paracrine mechanisms [79, 80]. In another study, transplantation after induced differentiation in vitro also significantly restored the dyskinesia of a 6-OHDA-treated PD mouse model [22]. To understand the mechanism of treatment, this study also determined that SHED secreted a large number of cytokines such as IL-6, GDNF, BDNF, and VEGF, which proved its immunomodulatory effect from the side. It is worth noting that the researchers added to the medium at the middle stage of neural induction the small molecule CHIR99021, which can inhibit the Wnt signal pathway of cells activated by GSK3β, thus increasing the production of DAergic neuron progenitor cells [81, 82]. Considering the survival rate of transplanted cells, a study used SHED-derived spheres instead of induced DAergic neurons for transplantation into a 6-OHDA-treated PD rat model [24]. Although it improved the dyskinesia of rats, the results were not very satisfactory. It may have been due to immune rejection, inflammatory response, correct integration of transplanted cells with the host brain, or for other reasons, which still needs to be further studied.

Based on the above studies, there may be three main mechanisms of SHED in the treatment of PD (Fig. 1). First, DAergic neurons or other neurons differentiated by SHED in vivo form a functional connection with the host neuron. Transplanted DAergic neuron-like cells can secrete DA to restore the functional activity of neurons. Second, the transplanted SHED can secrete cell growth factors, such as VEGF, BDNF, and GDNF. Some studies have reported that undifferentiated MSCs can secrete neurotrophic factors such as BDNF, GDNF, NGF, HGF, and VEGF to play a neuroprotective role [12, 83]. Third, through the immune regulation by cytokines such as IL-6 and TNF-α, these factors may combine with VEGF, BDNF, and GDNF to enhance immune regulation and reverse the damage to host neurons [84].

Like other approaches to stem cell transplantation, non-invasive cell therapy remains to be explored. Recently, some studies have suggested that the exosome of SHED may offer a new strategy for the treatment of PD. All cultured cell types can secrete exosomes, which carry a variety of proteins, RNA, lipids, and various metabolites [85, 86]. From the point of view of the treatment of PD, the transfer of exosomes has several advantages. First, they can cross the BBB to reach the brain without complicated neurosurgery [87]. Second, this technique is safer because it avoids the risks of cell transplantation, such as low survival rate, immune rejection, and malignant transformation. Finally, it is a relatively simple, stable, and controllable system which is suitable for large-scale clinical production [88]. So, this technique may have equal or even superior potential to treat PD as the use of SHED. A study demonstrated the neuroprotective effects of SHED-derived exosomes in vitro through ReNcell VM human neural stem cell lines, inhibiting about 80% of 6-OHDA-induced apoptosis [17]. In another study, the SHED exosomes were delivered into a 6-OHDA-treated PD rat model by intranasal administration, and it was found that the rat recovered from dyskinesia, and expression of TH in substantia nigra and striatum was normalized [21]. However, it has not been proven that exosomes increase the expression of TH in substantia nigra and striatum by directly affecting dopaminergic neurons or indirectly increase the expression of TH by regulating the response of astrocytes and microglia. It is also possible that both mechanisms operate at the same time. Taken together, these findings suggest that the use of exosomes in early preclinical studies may be a promising method for the treatment of PD.

## Clinical trials

In contrast to a large amount of evidence reported in basic studies, there is no clinical application of DPSCs or SHED in the clinical treatment of PD.

Many regenerative medicine studies have shown that DPSCs and SHED can provide a good therapeutic effect for a variety of diseases, including various central nervous system diseases such as spinal cord injury, stroke, retinal injury, Alzheimer’s disease, and peripheral nerve injury [89]. In addition, clinical studies on related diseases are also under way. Some research groups use autologous DPSCs to transplant into patients with dental pulp injury to promote pulp formation. Long-term follow-up monitoring shows that they can safely and effectively promote pulp regeneration, and no adverse reactions are observed [90–92]. In addition, a number of clinical trials have been carried out on bone regeneration, stroke, and diabetes [93, 94]. All in all, DPSCs and SHED are expected to become clinical-grade cells that can be widely used in the treatment of various diseases in the future.

The first clinical trial of cell transplantation for PD began more than 30 years ago, using DAergic neuronal precursor cells derived from human foetal midbrain tissue, which are still being used in ongoing TRANSEURO studies [9]. According to the investigation and statistics, the clinical projects involving stem cell therapy for PD under research in the world include work with MSCs (NCT03550183, NCT03684122), NSCs (NCT03309514, NCT02452723), ESCs (NCT03119636), and iPSCs [41]. In these studies, cells are delivered to patients by intravenous or intracerebral injection, and the transplanted cells play a neuroprotective role through neuronal differentiation or anti-inflammatory activity. The subjects have been followed up at different times to evaluate the safety and effectiveness of the treatment through various rating scales so as to provide a basis for further clinical research.

For DPSCs and SHED, first, they have the characteristics common to other stem cells. Second, these cells have unique advantages. (1) They have strong potential for neural differentiation because of the origin of their neural crest. (2) The ethical problems caused by transplantation can be avoided. (3) More importantly, these cells do not express costimulatory molecules, such as MHC-II, CD40, CD80, or CD86; therefore, they not only do not activate the immune system but also regulate immune rejection [95, 96]. Therefore, these cells are a good potential material among all the stem cell sources that can be used to treat PD.

Many studies demonstrated that just MSCs alone are not effective enough for neurodegenerative diseases and neurotrophin engineered MSCs are more effective [97, 98]. The engineering of dental stem cells aims to enhance specific functions to achieve stable therapeutic effects, including proliferation and neurogenic differentiation. In general, there are three common embellishments: (1) Differentiation in vitro. It is common to add neurotrophins such as EGF, bFGF, and BDNF to the differentiation medium in vitro to promote their maturation into DAergic neuron-like cells [99, 100]. Compared with undifferentiated cell transplantation, it can increase the level of DA and the secretion of neurotrophic factors more effectively and promote the recovery of neurological impairment [23]. (2) Genetic engineering. It is well known that DPSCs and SHED can secrete a variety of cytokines and nerve growth factors, such as BDNF and GDNF. Studies have shown that BDNF and GDNF have significant repair and protection effects on neurodegenerative diseases, including promoting axonal regeneration and reducing apoptosis [101, 102]. Therefore, some new treatments emerge as the times require, including the overexpression of BDNF and GDNF and the use of genetically engineered MSCs as a carrier to directly transfer cytokines to the microenvironment [97]. A team has tested the safety and efficacy of genetically engineered human MSCs in transgenic Huntington’s disease (HD) mouse models and published the results of new drug licensing [103]. Their results show that human genetically engineered MSCs can significantly reduce striatal atrophy in HD mice by about 50% compared with non-transgenic mice. In addition, it has been confirmed that MSCs transfected with GDNF can protect the nigrostriatal pathway from inflammatory Parkinson’s syndrome [98]. (3) Modification of chemical materials. The use of MSC-based nanostructured and microstructural materials for neurotrophic delivery is an effective way to improve the characteristics and therapeutic efficacy of stem cells, such as hydrogels and graphene [104–106]. These results suggest that it may be a promising direction to consider the engineering treatment of these cells in the future research on the treatment of PD with DPSCs and SHED.

## Conclusions

Recently, stem cell transplantation has attracted increasing attention as a treatment for neurodegenerative diseases. Many results have been achieved in the use of DPSCs and SHED in various in vitro and in vivo models of PD. This review summarizes their current research progress in the treatment of PD in some detail. Whether from the perspective of basic research or in clinical application, they have more advantages than do other stem cells and are a great choice for PD treatment. This insight has a profound enlightening effect for the further clinical research on PD. However, further research is warranted to study the mechanisms, immune rejection, survival rate, and delivery mode associated with this treatment.

## Data Availability

Not applicable.

## Abbreviations

BBB: Blood-brain barrier
BDNF: Brain-derived neurotrophic factor
bFGF: Basic fibroblast growth factor
BMP-2: Bone morphogenic protein 2
BMSCs: Bone marrow mesenchymal stem cells
CNP: 2′, 3′-Cyclic nucleotide 3′-phosphodiesterase
COMT: Catechol-O-methyltransferase
DA: Dopamine
DAergic: Dopaminergic
DBS: Deep brain stimulation
DPSCs: Dental pulp stem cells
ESCs: Embryonic stem cells
FGF: Fibroblast growth factor
GDNF: Glial cell line-derived neurotrophic factor
GFAP: Glial fibrillary acidic protein
HD: Huntington’s disease
iPSCs: Induced pluripotent stem cells
MAO-B: Monoamine oxidase B
MPTP: 1-Methyl-4-phenyl-1,2,3,6-tetrahydropyridine
MSCs: Mesenchymal stem cells
NGF: Nerve growth factor
NSCs: Neural stem cells
NSE: Neuron-specific enolase
PD: Parkinson’s disease
SCI: Spinal cord injury
SHED: Stem cells from human exfoliated deciduous teeth
SNpc: Substantia nigra pars compacta
TH: Tyrosine hydroxylase
6-OHDA: 6-Hydroxydopamine
